# Reduced white matter venous density on MRI is associated with neurodegeneration and cognitive impairment in the elderly

**DOI:** 10.3389/fnagi.2022.972282

**Published:** 2022-09-01

**Authors:** Chenyang Li, Henry Rusinek, Jingyun Chen, Louisa Bokacheva, Alok Vedvyas, Arjun V. Masurkar, E. Mark Haacke, Thomas Wisniewski, Yulin Ge

**Affiliations:** ^1^Department of Radiology, Center for Biomedical Imaging, NYU Grossman School of Medicine, New York, NY, United States; ^2^Vilcek Institute of Graduate Biomedical Sciences, NYU Grossman School of Medicine, New York, NY, United States; ^3^Department of Psychiatry, NYU Grossman School of Medicine, New York, NY, United States; ^4^Department of Neurology, NYU Grossman School of Medicine, New York, NY, United States; ^5^Department of Radiology, Wayne State University School of Medicine, Detroit, MI, United States; ^6^Departments of Pathology, NYU Grossman School of Medicine, New York, NY, United States

**Keywords:** neurodegeneration, venous density, cognitive impairment, susceptibility weighted image (SWI), venous oxygenation

## Abstract

High-resolution susceptibility weighted imaging (SWI) provides unique contrast to small venous vasculature. The conspicuity of these mesoscopic veins, such as deep medullary veins in white matter, is subject to change from SWI venography when venous oxygenation in these veins is altered due to oxygenated blood susceptibility changes. The changes of visualization in small veins shows potential to depict regional changes of oxygen utilization and/or vascular density changes in the aging brain. The goal of this study was to use WM venous density to quantify small vein visibility in WM and investigate its relationship with neurodegenerative features, white matter hyperintensities (WMHs), and cognitive/functional status in elderly subjects (*N* = 137). WM venous density was significantly associated with neurodegeneration characterized by brain atrophy (β = 0.046± 0.01, *p* < 0.001), but no significant association was found between WM venous density and WMHs lesion load (*p* = 0.3963). Further analysis of clinical features revealed a negative trend of WM venous density with the sum-of-boxes of Clinical Dementia Rating and a significant association with category fluency (1-min animal naming). These results suggest that WM venous density on SWI can be used as a sensitive marker to characterize cerebral oxygen metabolism and different stages of cognitive and functional status in neurodegenerative diseases.

## Introduction

Age-related cognitive and functional decline are increasingly prevalent due to the rise in Alzheimer’s disease (AD) and related dementias (Alzheimer’s Association, 2020). Varying degrees and patterns of brain atrophy have been reported in different types of dementia such as AD, Parkinson’s disease, frontotemporal lobar degeneration, and vascular dementia (Frisoni et al., 1996; Burton et al., 2004; Fox and Schott, 2004; Logue et al., 2011). Despite an unclear mechanism, brain atrophy assessed using volumetric imaging has been used to detect the disease onset, monitor its progression, and differentiate among dementia subtypes (Risacher et al., 2017). Unfortunately, by the time brain atrophy can be measured on volumetric imaging, the patient may already be suffering from significant cognitive decline and thus have few treatment options. Studies of ^18^F-FDG-PET have shown that decreased glucose metabolism in AD brain can be detected long before brain atrophy or clinical symptoms become apparent (Mosconi, 2005). It revealed that the reduction of glucose metabolism of neuronal cells is not due entirely to atrophy but reduced utilization per gram of tissue in AD (Ibanez et al., 1998). However, the high cost and limited availability of PET imaging make it less than ideal for routine monitoring of a rapidly aging population. So far, one of the most challenging aspects of age-related dementia remains the early detection of neurodegenerative pathophysiology, including a more accessible early imaging marker of neurodegeneration before structural volume loss or symptoms occur.

Susceptibility-weighted imaging (SWI) is an established clinical imaging sequence for examination of the venous system and microbleeds in the brain (Sehgal et al., 2005; Mittal et al., 2009). SWI, which relies on paramagnetic deoxygenated hemoglobin as intrinsic T2* contrast agent (Reichenbach et al., 1997; Haacke et al., 2004), enhances the visibility of the brain’s vasculature and enables quantitative estimates of the venous density (Vigneau-Roy et al., 2014; Bernier et al., 2018; Buch et al., 2021). SWI provides not only rich information about the anatomy of the venous vasculature, including small veins, but also an assessment, albeit indirect, of oxygen metabolism of the brain *via* unique venous blood susceptibility contrast of veins with the background tissue (Ge et al., 2009). Recent studies have demonstrated that SWI is highly sensitive to changes of venous oxygenation level caused, for example, by ingesting caffeine, breathing carbogen, or even by simple alteration of respiratory pattern (Sedlacik et al., 2008; Chang et al., 2014). In brain tissue with impaired neuronal metabolism, unconsumed oxygen drains directly into the veins, thus elevating the level of diamagnetic oxygenated hemoglobin in the veins. This, in return, alters the venous T2* and lowers the phase difference between the veins and surrounding tissue (Li et al., 2013). As a result, small veins become less conspicuous on SWI images. The very opposite occurs in stroke when there is perfusion deficit creating an enhancement of the veins in areas of reduced perfusion (Xia et al., 2014; Rastogi et al., 2015; Lu et al., 2021).

In this study, we used WM venous density as a semi-quantitative estimate to assess the visibility of the periventricular small veins’ appearance on SWI. To characterize if WM venous density can be used as a potential marker of neuronal oxygen metabolism, we examined the relationship of WM venous density to neurodegenerative features (e.g., gray matter volume fraction and parenchymal volume fraction) and cerebral microangiopathy (e.g., WMHs) as well as cognitive performance metrics in elderly individuals.

## Materials and methods

### Study participants

The imaging protocol was approved by the Institutional Review Board at New York University (NYU) Grossman School of Medicine. In this HIPAA-compliant and IRB-approved cross-sectional study, we examined consecutive baseline MRI of 137 elder participants at the NYU Langone Health’s Alzheimer’s Disease Research Center (NYULH-ADRC). Imaging took place between March 2014 and December 2019. All participants gave written, informed consent before obtaining an MRI scan. Subjects with conditions that could have confounded the interpretation of venous density were excluded, including any significant neurologic disease (other than AD) such as Parkinson’s disease, Huntington’s disease, infarction, normal pressure hydrocephalus, brain tumor, progressive supranuclear palsy, seizure disorder, subdural hematoma, multiple sclerosis, and head trauma. Also excluded were subjects that had contraindications for MRI studies, including the presence of metal or foreign objects in the eyes, skin or body.

### Cognitive assessments

All NYU-ADRC subjects underwent a detailed neurocognitive assessment as defined by the National Alzheimer’s Coordinator Center’s Uniform Data Set. This included Clinical Dementia Rating (CDR^®^, henceforth CDR) and the One-Minute Animal Test (OMAT) score. The CDR is a widely utilized tool for grading the presence and severity of dementia (Morris, 1993; O’Bryant et al., 2008), based on a five-point scale (0, 0.5, 1, 2, 3) that assesses the characteristics of cognitive impairment and function across 6 domains. The standard global CDR score ranges from 0 (normal), 0.5 (mild cognitive impairment or MCI), and 1–3 (increasing severity of dementia). Alternatively, sum-of-boxes of the CDR (CDR-sum) is the sum of all the elemental components across the 6 categories, (range 0–18) providing a more granular range to define the degree of cognitive and functional impairment. The OMAT is a fast assessment to measure semantic fluency by asking the subject to list as many animals as possible in 1 min; a low OMAT score (≤ 13) indicates a symptom of dementia (Kinuhata et al., 2018).

### MRI protocols

All subjects were scanned under a clinical MRI protocol including: T1-magnetization prepared rapid gradient echo (T1-MPRAGE), T2-fluid attenuated inversion recovery (T2-FLAIR) and high in-plane resolution SWI sequence on a 3 Tesla (3T) MRI system (Siemens Prisma) using a 64-channel head coil. MPRAGE images were acquired using the following parameters: TE/TR: 5 ms/2,100 ms; matrix size: 256 × 256 × 176; voxel size: 1 mm isotropic. T2-FLAIR images were acquired using the following parameters: TE/TR: 75 ms/9,000 ms; matrix size: 320 × 320 × 42; voxel size: 0.6875 mm × 0.6875 mm × 4 mm. High in-plane resolution flow-compensated SWI images were acquired using gradient-echo sequence using following parameters: TE/TR: 25 ms/50 ms; matrix size: 512 × 512 × 32; voxel size: 0.4297 mm × 0.4297 mm × 1.5 mm. All scans were collected parallel to the anterior commissure to posterior commissure (AC-PC) line. The 48 mm SWI slab covered the entire periventricular region with the lateral ventricle in the center of the slab.

### Quantification of white matter hyperintensities volume and fractional volume indices

Absolute volume of white matter hyperintensities was estimated from T2-FLAIR images using an automated bilateral distance partitioning method (Chen et al., 2021) applied to total WMHs masks (obtained using FireVoxel software^1^) to yield periventricular WMHs lesion (Pv-WMHs) mask. Total WMHs and Pv-WMHs lesion volume were estimated based on the mask and its voxel size. We used the FSL FMRIB’s Automated Segmentation Tool *FAST* segmentation toolbox on the T1-MPRAGE data to yield a probability mask and a binary mask of three tissue types including white matter (WM), gray matter (GM) and cerebrospinal fluid (CSF). Brain parenchymal fraction (BPF), gray matter fraction (GMF) were calculated as the volumetric ratio of brain parenchyma (WM and GM total volume), gray matter to the intracranial volume (WM, GM and CSF total volume), which were then used as a volume index to characterize neurodegeneration.

### Quantification of venous density

**FIGURE 1:**
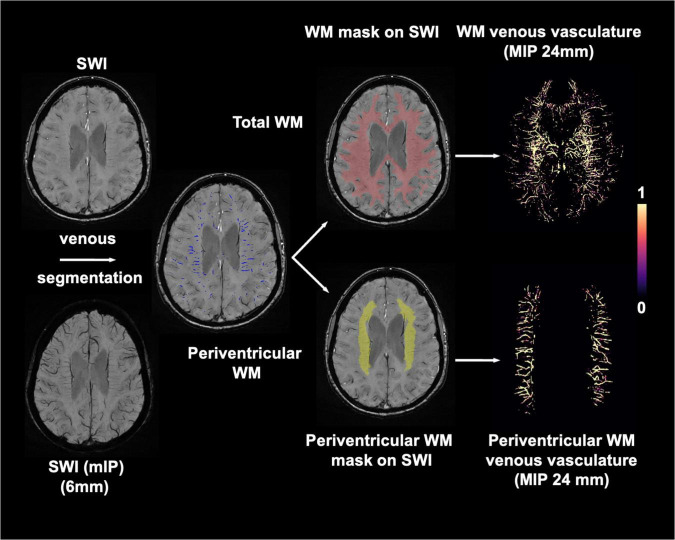
Imaging processing pipeline for extracting white matter (red) and periventricular white matter (yellow) venous vasculature using preprocessed SWI and T1-MPRAGE images. Maximum intensity projection (MIP) of venous masks is shown for visualization.

SWI images were preprocessed with 1) bias field uniformity correction and 2) de-noising using a spatially adaptive non-local means algorithm *N3BiasFieldCorrection* and *DenoiseImage* commands in ANTs^2^ to avoid potential inaccurate segmentation from noise and field inhomogeneity on SWI images. MPRAGE and T2-FLAIR volumes were co-registered to SWI images using *FLIRT* linear registration in FSL. Then white matter and WMHs mask generated from T1-MPRAGE and T2-FLAIR images were spatially transformed to SWI space. Venous segmentation was performed on preprocessed SWI with a novel vascular segmentation toolbox developed by Bernier et al.^3^ using multiple iterative Frangi filter and vessel enhancing diffusion (VED) filter to extract tubular shapes of different size as vessels. A more detailed description of the segmentation algorithm can be found in Bernier et al. (2018). WM venous density was calculated as volumetric fraction of veins in white matter on SWI images. To evaluate the global and regional white matter venous density, the periventricular white matter region was extracted from the T1-MPRAGE data using an automatic multi-atlas based segmentation tool (MRIcloud, Johns Hopkins University, MD, Baltimore^4^) (Wu et al., 2016). The segmentation was based on pre-defined elderly brain atlases (age between 50 and 90 years) with 289 partitions that include periventricular white matter structures where most of the periventricular WMHs and deep medullary veins are located. The periventricular white matter density was calculated using the same approach as white matter venous density. Finally, a scaled venous vasculature map in each region was created. The flowchart of the image processing pipeline is illustrated in Figure 1. To prevent false positive segmentation caused by hypointense regions other than veins, microbleeds and susceptibility artifacts near the sinuses were manually excluded from the WM mask on SWI images by an experienced neuroradiologist (YG 20 years of experience).

### Statistical analysis

Statistical analysis was performed using Graphpad Prism (Graphpad, San Diego, CA^5^) and Matlab R 2019b (MathWorks, Natick, MA, 2019). One-way analysis of variance (ANOVA) was used to characterize the difference in age, sex, education and WM venous density among three groups with different CDR-sum score. The Bonferroni correction was used to adjust for multiple comparisons. Multiple linear regression was used to examine the relationship between WM venous density (dependent variable) and estimates of neurodegeneration: GMF and BPF. Linear regression was also performed to investigate the association of WM venous density to CDR-sum and OMAT score. We also examined the association of WM venous density and periventricular WM venous density with total WMHs lesion and periventricular WMHs lesion volumes. In this model, WM/Pv-WM venous densities were dependent variables and WMHs/Pv-WMHs lesion volumes were independent variables. All linear regression analyses were adjusted for age, sex and education as covariates for a two-tailed *p*-value below 0.05 (*p* < 0.05) which was considered as statistically significant. The *p* values for regression analyses were also tested with False Discovery Rate (FDR) to evaluate statistical significance.

## Results

### Subjects characteristics

In total, 137 elderly participants (age, 71.1 ± 9.5 years; 32 males and 105 females; education, 16.8 ± 2.9 years) were included in this study. The subject characteristics are shown in Table 1. The majority of subjects (64%) were cognitively normal (CDR = 0), 35% were MCI (CDR = 0.5), and 1% had increasing dementia symptoms (CDR≥ 1). The MRI data from 137 subjects were used for volumetric analysis on neurodegeneration and WMHs. For the cognitive performance analysis, six subjects were excluded because of an incomplete CDR assessment, and four subjects lacked the OMAT results. Thus, 131 subjects were used to evaluate the association between SWI and cognitive metrics.

**TABLE 1:** Characteristics of participants.

	Total	CDR = 0	CDR = 0.5	CDR = 1
Subjects number (N)	137 (131*)	84	46	1
Age (years)	71.1 ± 9.5	69.8 ± 9.4	74.9 ± 7.7	82.7
Sex (female/male)	105/32 (30**)	64/20	36/10	1/0
Education (years)	16.8 ± 2.9	17.1 ± 2.7	16.7 ± 2.3	20

*Six participants in total lack of CDR-global score. **Two of the male participants lack of CDR-global score. All data are presented as mean standard deviation.

### Visibility of white matter venous vasculature in the presence of neurodegeneration

**FIGURE 2:**
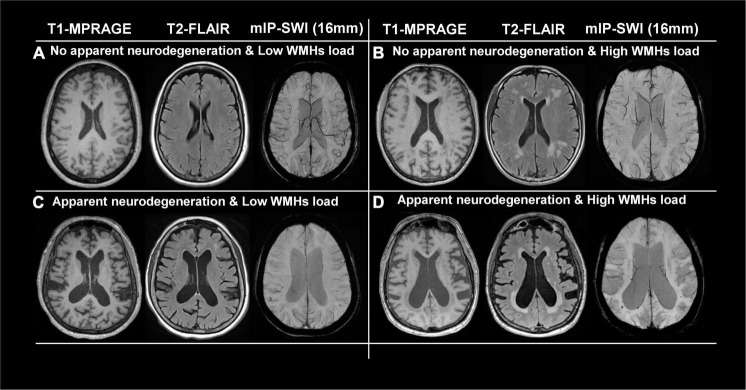
Representative images of T1-MPRAGE, T2-FLAIR, and minimum intensity projection (mIP) of SWI images of four representation groups: **(A)** with no apparent neurodegeneration and low WMHs load; **(B)** with no apparent neurodegeneration and high WMHs load; **(C)** with apparent neurodegeneration and low WMHs load; and **(D)** with apparent neurodegeneration and high WMHs load.

**FIGURE 3:**
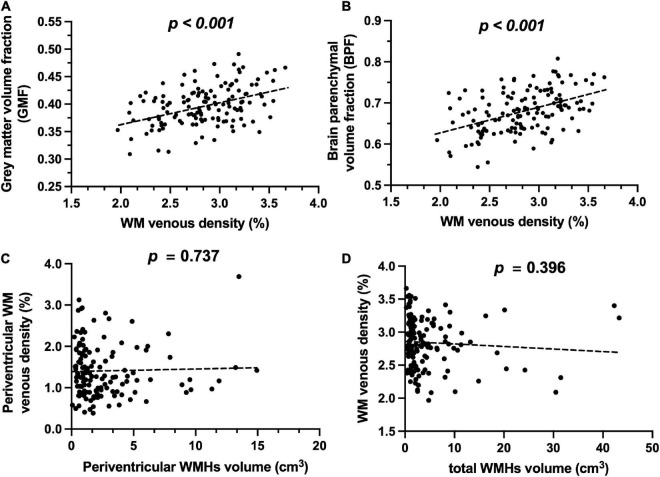
Relationship of WM venous density to GMF, BPF, and WMHs. **(A)** Scatter plot between WM venous density and GMF. **(B)** Scatter plot between WM venous density and BPF. **(C)** Scatter plot between periventricular WM venous density and periventricular WMHs load. **(D)** Scatter plot between WM venous density and total WMHs load.

Figures 2A,C show representative T1-MPRAGE, T2-FLAIR and minimum-intensity projected SWI (mIP-SWI) of subjects without and with apparent neurodegeneration. Low visibility of deep medullary veins was observed in the setting of severe neurodegeneration in GM and WM. Figures 3A,B show the association of WM venous density with fractional volume indices; that is, WM venous density is positively associated with BPF (β = 0.046± 0.01, *p* < 0.001), GMF (β = 0.037± 0.01, *p* < 0.001).

### Visibility of white matter venous vasculature in the presence of white matter hyperintensities

**FIGURE 4:**
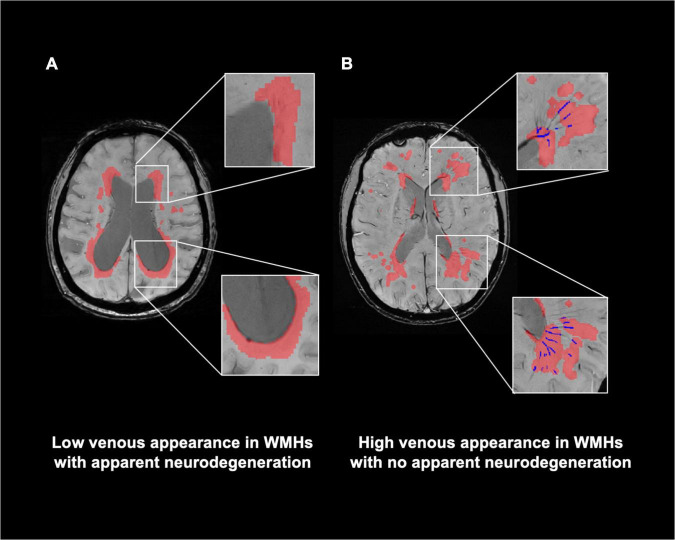
Relationship of visibility of small veins to WMHs. **(A)** Representation of WMHs lesion (red) on SWI image of a patient with apparent neurodegeneration. **(B)** SWI image with delineation of WMHs and veins within WMHs (blue) of a patient with no apparent neurodegeneration. Conspicuous small veins were delineated penetrating or surrounding WMHs when patients have no apparent neurodegeneration, whereas patients with apparent neurodegeneration have lower visibility of small veins.

Figures 4A,B illustrate representative cases (shown in Figures 2B,D) of SWI images overlaid with the WMHs lesion mask. Conspicuous small veins were delineated penetrating or surrounding WMHs when there was no apparent neurodegeneration, whereas with subjects showing apparent neurodegeneration there was lower visibility of small veins. In addition, quantitative analysis revealed no significant association of white matter venous density with WMHs lesion load (*p* = 0.396). We also examined the association of Pv-WM venous density with Pv-WMHs lesion load and no association was found (*p* = 0.739).

### Relationship of white matter venous density with cognitive evaluation

**FIGURE 5:**
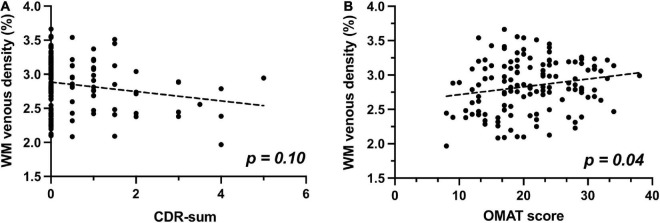
Relationship of WM venous density to clinical cognitive evaluations. **(A)** Scatter plot between WM venous density and CDR-sum. **(B)** Scatter plot between WM venous density and OMAT test score.

When examining the association between WM venous density with cognitive evaluations, as demonstrated in Figure 5A, there is a trend of negative association of WM venous density with CDR-sum score (*p* = 0.101). We further investigated the relationship of WM venous density to OMAT test scores (*p* = 0.040). In Figure 5B, it showed a significant positive association with WM venous density.

## Discussion

In this study, we quantitatively evaluated the venous oxygen saturation dependent visibility of WM venous vasculature on high resolution SWI, in a cohort of elder individuals. In AD, neurodegeneration underlies both reduced cell activity in early stages and cell loss in relatively late stages. SWI can provide insight into the early detection of neurodegeneration, *via* its ability to detect reduced oxygen metabolism. Using SWI, qualitative and quantitative assessment of venous oxygen saturation has never been so feasible. It can directly visualize small veins with high sensitivity in detecting different levels of (de)oxygenated hemoglobin without radiotracers. Previous studies have shown the change of venous contrast in SWI venography when the venous oxygen level is altered (Rauscher et al., 2005). Unlike other imaging techniques that measure oxygen metabolism in the brain including ^15^O PET (Mintun et al., 1984) and T2- or R2-based MRI (Xu et al., 2009; Mao et al., 2018), SWI is routinely used in clinical evaluation of neurological and psychological disorders thanks to its clinical accessibility and fast acquisition (e.g., < 5 min) on most scanners.

Compared to large venous structures, such as internal cerebral veins, the contrast of WM small veins on SWI venography are more sensitive to the changes of deoxygenated hemoglobin level. Thus, we investigated whether WM venous density could quantitatively depict the oxygen metabolism in aging brains and different stages of cognitive impairment. In elderly populations, neurodegeneration and WMHs are two common radiological markers seen in age-related diseases such as AD and other types of dementia. We found strong correlations between WM venous density and fractional volume indices such as BPF and GMF, suggesting subjects with a higher degree of neurodegeneration in both gray and white matter have lower WM venous visibility since the neurodegenerative features of brain structures would contribute to higher unconsumed oxygenated level in WM small veins.

Previous studies have shown the reduced venous visibility and venous density on SWI images in multiple sclerosis (MS) patients, indicating the potential alteration of neuronal metabolism caused by neurodegeneration in MS (Ge et al., 2009; Zivadinov et al., 2011; Sinnecker et al., 2013), which is consistent with findings using a quantitative measure of cerebral metabolic rate of oxygen (Ge et al., 2012). Furthermore, veins in white matter collect blood from both white matter and gray matter. Thus, WM venous density may be a potential target to describe overall metabolic changes caused by neurodegeneration from both tissue structures. Loss of brain volume likely indicates the damage and loss of neural processing and computational components such as neurons and synapses, which are thought to be major energy-consuming components in the brain. Unconsumed arterial blood will increase the concentration of oxygenated hemoglobin in small veins, leading to altered T2* decay in the veins and reduced the phase difference between veins and tissue, or reduced “blooming effect” that decrease the sensitivity in depicting small veins. On the other hand, physical loss of venous structures at mesoscopic level is usually concomitant with other MR-visible pathologies such as venous thrombosis or microbleeds. Thus, we conclude that the contrast changes of small veins on SWI are mainly coming from altered level of tissue venous oxygenation caused by neurodegeneration instead of vessel degradation. These findings underline the importance of SWI being a potential imaging marker of neurodegeneration for designing and interpreting future neuroprotective treatment trials.

Despite there being a strong correlation between WM venous density and fractional volume indices, we did not find a correlation between WM venous density and total WMHs load (Figure 3D). Regional analysis of the periventricular territory also revealed no significant correlation between periventricular WM venous density and Pv-WMHs (Figure 3C). As Figures 2, 4 show, regardless of the presence of WMHs, the WM venous appearance was largely diminished with severe neurodegeneration, while with higher WMHs load and without apparent neurodegeneration, conspicuous venous visibility of small veins (diameter 50-400 μ m) was still observed within WMHs lesions. Our finding is consistent with other studies that WMHs do not have significant effect on overall metabolic rate of the brain (Thomas et al., 2017; Jiang et al., 2020). On one hand, high visibility of venous vasculature in WMHs may indicate an elevated level of deoxygenated hemoglobin in WMHs region. Although the etiology of WMHs is still not fully understood, growing evidence shows that WMHs may still be salvageable tissue of ischemia origin (Prins and Scheltens, 2015). This hypoxia metabolic stress may lead to higher oxygen extraction in WMHs lesions in order to maintain appropriate neural activity. As a result of increased level of deoxygenated hemoglobin in the veins in WMHs with enhanced visibility, Yan et al. (2014) showed that increased visibility of deep medullary veins in leukoaraiosis. Kesavadas et al. (2010) also found that in carotid stenosis, the extremely reduced cerebral blood flow (CBF) to the brain could lead to the enhancement of the venous contrast on the ipsilateral side of the insulted brain. On the other hand, physical changes of venous structures, such as periventricular venous collagenosis (Moody et al., 1995; Keith et al., 2017), may be another factor that could alter the venous appearance due to its association with hypertension (Zhou et al., 2015) and disrupted venous functions (Haacke et al., 2021).

From an anatomical perspective, the venous vasculature within the WMHs lesion does not drain blood solely from WMHs, it also drains from subcortical gray matter and deep white matter regions. The venous pathways could just trespass into the WMHs. More importantly, in aging brains with different severity of neurodegeneration, both total WMHs and Pv-WMHs were not significantly correlated with venous density measures. The reduction in oxygen utilization caused by neurodegeneration may play a more dominating role compared to oxygen metabolism changes in WMHs. As WMHs are one of the typical microangiopathy findings in the elderly, they could provide insight in better understanding the pathologic pathway between neurodegeneration and vascular degeneration in AD and cognitive impairment (Haight et al., 2013; Kalaria and Ihara, 2013). In addition, vascular risk factors such as hypertension, diabetes and hypercholesterolemia are assumed to be linked with lower CBF. As a consequence of reduced perfusion, the concentration of deoxygenated hemoglobin may be different with low vascular risk populations and may result in altered contrast of SWI venography. As lower CBF often co-exists with neurodegeneration in aging brains, our results could provide a clue that the venous oxygenation level changes caused by neurodegeneration may outweigh the venous density changes due to reduced blood flow.

Findings from our study also suggest associations of WM venous density with clinical parameters. Although there is no significant correlation, we observed that there is a positive trend of WM venous density to CDR sum of boxes score, indicating the potential alteration of oxygenation level with different degrees of cognitive and functional impairment. Previous studies such as Jiang et al. (2020) even found an elevated venous oxygenation in dementia without vascular risk factors. Thus, WM venous density may offer an alternative approach and provide insight into changes of oxygen utilization in various disease stages of MCI and dementia if a clear etiology is specified.

In addition, we observed a significant association of WM venous density to the OMAT score, which is one of the most common and sensitive methods to test semantic fluency, and as a measure for characterizing cognitive processing speed. Previous studies that use functional near-infrared spectroscopy reported decreased hemodynamic responses in MCI and AD patients (Arai et al., 2006; Metzger et al., 2016; Katzorke et al., 2018), revealing the change of pattern in neural activity during verbal task. In addition, recent studies revealed that semantic fluency is an efficient method for early dementia screening (McDonnell et al., 2020) and semantic loss is correlated with AD-related neurodegeneration and deteriorates faster in early stage of AD (Vonk et al., 2020). Therefore, reduced WM venous density may denote the reduction in oxygen utilization caused by reduced neural activity in patients with a low OMAT score. The clinical cognitive results suggest that WM venous density may be used as a novel marker for venous oxygenation changes related to neural activity in aging brains at different stages, even prior to overt brain atrophy.

Several limitations should be noted in this study. First, WM venous density is a less direct approach to quantitatively describe the susceptibility difference in the veins compared to quantitative susceptibility map (QSM), which would yield venous susceptibility value. However, reconstruction of venous susceptibility QSM has yet been standardized in clinical applications, which still requires optimization in the data processing pipeline for quantitative accuracy (Berg et al., 2021). The major difficulty in using QSM is the partial volume effect of small veins which makes oxygen saturation quantification suspect. Therefore, the appropriate technique is required to validate the consistency between venous density and QSM approaches for future clinical applications. Second, venous density can only be performed on a regional basis. However, it is challenging to evaluate the venous density within the WMHs lesions, as there is a large variation in the total WMHs lesion load, making the quantification of WMHs-specific venous density less consistent across all subjects. Third, the lack of amyloid and tau biomarkers to define the cohort may limit the results interpretation specifically to the AD spectrum. Fourth, characterization of CBF, which could help interpretation of our results, was not included in this study. Finally, the sex difference is not compared for this study due to the unmatched numbers of male and female participants. The hematocrit difference in male and female may lead to different oxygenation levels. Therefore, sensitivity of a gender-specific difference measured by SWI venography needs further evaluation.

## Conclusion

Our study showed that reduced venous density on SWI is associated with neurodegeneration characterized by fractional volume indices in elderly brains, suggesting that a SWI venous density measure could be used to monitor age-related neurodegeneration. Diminished small vein visibility could be associated with reduced oxygen utilization in neurodegenerative structures but may be minimally altered in regions where WMHs present. Furthermore, we demonstrated that reduced WM venous density is associated with cognitive and functional impairment and subjects’ increasing impairment showed lower WM venous density.

## Data availability statement

The original contributions presented in this study are included in the article/supplementary material, further inquiries can be directed to the corresponding author/s.

## Ethics statement

The studies involving human participants were reviewed and approved by New York University Grossman School of Medicine, Institutional Review Board. The patients/participants provided their written informed consent to participate in this study.

## Author contributions

CL and YG conceived, designed, and wrote the manuscript. CL, JC, and HR contributed to the methodology and data analysis. All authors contributed to manuscript revision, proofreading and approval prior to the final submission.

## Footnotes

1
https://firevoxel.org

2
http://stnava.github.io/ANTs/

3
https://github.com/braincharter/vasculature

4
www.MRICloud.org

5
www.graphpad.com
